# In this month’s *Bulletin*

**DOI:** 10.2471/BLT.15.001015

**Published:** 2015-10-01

In the editorial section, Peter Ventevogel et al. (666) call for the provision of mental health care in humanitarian emergencies. Brien A Holden et al. (667) emphasize the importance of better global estimates of presbyopia.

In the news section, Dale Gavlak reports on health workers’ struggles to help civilians caught up in the conflict in Yemen (670–671). Rodrigo Guerrero-Velasco tells Alyssa Greenhouse how violence can be understood and prevented using an epidemiological approach (672-673). 

## Argentina, Brazil, Colombia, Chile, Mexico and Peru

### Pivotal trials and access-to-medicines

Núria Homedes & Antonio Ugalde (674–683) study the availability of new medicines in the countries where they were tested.

## Bangladesh

### As bad as smoking?

Fen Wu et al. (684–692) estimate deaths due to betel quid use.

## Ethiopia

### Monitoring hospital performance

Zahirah McNatt et al. (719–726) describe a national system for assessing the quality of facility-based care.

## Jordan

**Providing better mortality data**

Faris Dababneh et al. (727–731) review the progress made in national mortality surveillance and reporting.

## Zambia

### Improving paediatric asthma care 

Somwe Wa Somwe et al. (732-736) describe a public-private partnership designed to improve the recognition and treatment of asthma. 

## Global 

### Values at stake in security agenda 

Lisa Eckenwiler et al. (737–738) discuss the unintended health impacts of counterterrorism policies and practices. 

### Treating paediatric tuberculosis

Meaghann S Weaver et al. (700–711) analyse the design, delivery and outcomes of interventions to improve treatment adherence in low-resource settings. 

### Selecting essential medicines

Corrina Moucheraud et al. (693–699) evaluate the quality of economic data in applications to the WHO Model List of Essential Medicines.

### Eliminating podoconiosis

Kebede Deribe et al. (712-718) propose case definitions and elimination targets for endemic non-filarial elephantiasis.

### Antenatal care in prisons 

Molly Skerker et al. (739-740) describe obstacles to providing care to incarcerated pregnant women. 

